# MR-guided laser interstitial thermal therapy for drug-resistant lesional epilepsy: a single-center experience

**DOI:** 10.1186/s41016-023-00335-2

**Published:** 2023-09-18

**Authors:** Hongchuan Niu, Kai Li, Xiaoning Liang, Desheng Kong, Zongze Li, Fengqiao Sun, Xianzeng Liu, Zongsheng Xu, Xuejiao Wei, Shuang Lan, Changyu Lu

**Affiliations:** 1 Department of Neurosurgery, Peking University International Hospital, Beijing, China; 2 Department of Neurosurgery, PKUCare Zibo Hospital, Shandong, China; 3 Department of Neurology, Peking University International Hospital, Beijing, China; 4 Present Address: Department of Neurosurgery, Peking University International, No.1 Shengmingyuan Road, Zhongguancun Life Science Park, Changping District, Beijing, 102206 China

**Keywords:** Laser, MRgLITT, Epilepsy

## Abstract

**Background:**

To describe and report the efficacy and safety of MR-guided laser interstitial thermal therapy (MRgLITT) in the treatment of drug-resistant epilepsy.

**Methods:**

A retrospective review of all MRgLITT procedures in our hospital was performed. All procedures were performed using a surgical laser ablation system. Demographic and outcome data were compiled and analyzed.

**Results:**

A total of 19 patients underwent MRgLITT procedures from June 2021 to November 2021. The average age at surgery was 18.1 years (3–61.4 years). The average length of hospitalization post-surgery was 4.95 days (4–7 days). Surgical substrates included 8 patients with hypothalamic hamartomas, 5 with medial temporal lobe epilepsy, 3 with deep focal cortical dysplasia, 1 with tuberous sclerosis, 1 with a cavernous malformation, and 1 with Lennox–Gastaut syndrome who underwent anterior corpus callosotomy. Complications occurred in three patients. After an average follow-up of 1 year, 6 patients were seizure-free (Engel I, 31.6%), 1 had significant seizure control (Engel II, 5.3%), 7 had seizure control (Engel III, 36.8%), and 5 had no improvement in their seizures (Engel IV, 26.3%). Fisher’s exact tests did not reveal statistical significance for the association between Engel class outcome and epileptic disease.

**Conclusion:**

This study confirmed that MRgLITT, as a method for treating drug-resistant epilepsy, is minimally invasive, safe, and efficient and that it can reduce the incidence of surgery-related complications.

## Background

Seizures affect 10% of the world’s population and cause 1–2% of the world’s population to suffer from epilepsy. Epilepsy is one of the most common neurological diseases, affecting approximately 50 million people worldwide, with an annual incidence rate ranging from 50.4 to 81.7 per 100,000 people. The prevalence of epilepsy is likely to increase because more people are surviving serious head trauma, stroke, and intracranial infections and living longer with primary and secondary brain tumors than ever before [1]. Antiepilepsy drug treatment is ineffective for nearly 30% of epilepsy patients. For these patients, surgical treatment is becoming a more viable option. Although surgery has traditionally been used as a palliative therapy, advancements in technology and results have demonstrated its potential in certain patient subgroups [2].

While laser ablation for tumors was first reported in 1980, Sugiyama et al. first reported stereotactic laser ablation for brain tumors in 1990, and Curry et al. reported the use of laser interstitial thermal therapy (LITT) for epileptic lesion ablation in 2012. Currently, an increasing number of epilepsy treatment centers are using LITT to treat epilepsy because it has been proven to be effective in a number of research trials [2]. The first magnetic resonance (MR)-guided LITT (MRgLITT) system for use in the brain soft tissue was approved by the US Food and Drug Administration (FDA) in 2007. Based on the results of these trials, we believe that this technology is a minimally invasive, safe, and effective method for treating epilepsy.

## Methods

### Research design

From June 2021 to November 2021, patients who received MRgLITT treatment using a surgical laser ablation system were studied retrospectively. All of the procedures were performed at our hospital. This study was approved by our hospital’s drug clinical trial center’s research ethics review committee. All patients or their guardians signed informed consent forms prior to the surgery. Demographic, intraoperative, and outcome data were collected and analyzed. We simultaneously collected the preoperatively planned ablation range and the actual postoperative ablation range and calculated the intersection volume to calculate the ablation rate and accuracy (Table 1).

**Table 1:** Demographic, perioperative, and outcome data

Patient	Gender	Age, years	Surgical substrate	AEDs	Lesions ablated, *n*	Blood loss, ml	Planned ablation volume, mm^3^	Actual ablation volume, mm^3^	Intersection volume, mm^3^	Ablation rate	Hospital stay, days	Outcome(Engel class)	Complications	Prior surgery
1	M	5.4	HH	LEV	1	1	659.164	759.414	610.284	92.6%	4	III	None	Craniotomy
2	M	4.9	HH	OXC	1	10	1667.92	1722.65	1537.59	92.2%	4	III	None	None
3	M	6	HH	LTG, LCM	1	5	485.148	606.796	406.62	83.8%	5	III	None	Craniotomy
4	F	6.2	HH	LEV, OXC	1	5	755.432	841.078	690.878	91.5%	4	I	None	RF-TC
5	M	19.5	HH	LTG, OXC	1	5	153.248	208.446	141.615	92.4%	4	III	None	Craniotomy
6	M	35.2	MTLE	OXC, LTG, VPA	1	10	3259.42	3764.87	3066.86	94.1%	4	I	None	None
7	F	24.6	MTLE	LEV, VPA	1	20	3046.08	3310.68	2788.99	91.6%	4	IV	Visual field defect	Craniotomy
8	M	15.1	MTLE	LTG, OXC	1	20	3516	3548.85	3204.59	91.1%	6	IV	None	None
9	M	3	HH	TPM	1	5	349.536	463.269	324.989	93%	5	IV	None	RF-TC
10	M	3.3	HH	LEV, VPA, LCM	1	5	1307.26	1515.17	1243.11	95.1%	5	III	None	RF-TC
11	M	3.2	HH	VPA	1	5	1845.45	2141.41	1695.22	91.9%	5	I	None	None
12	M	36.9	FCD	CBZ, LTG, VPA, TPM	1	2	4698.78	6582.23	4310.23	91.7%	7	I	Intracranial hemorrhage	Craniotomy
13	M	3.11	TSC	OXC, VPA	2	5	5627.49	6212.61	5323.48	94.6%	4	I	None	Craniotomy
14	F	61.4	CM	CBZ	1	5	1747.63	2130.37	1683.97	96.4%	4	I	None	None
15	F	10.2	FCD	LEV, OXC, LCM, PB	1	10	6033.22	7416.95	5513.87	91.4%	6	II	None	RF-TC
16	F	30.5	LGS(Corpus callosotomy)	TPM, CZP, LEV	1	10	2404.08	3353.268	2187.895	91.0%	7	IV	None	None
17	M	30.6	MTLE	OXC	1	5	4337.1	4603.68	3984	91.9%	4	IV	None	RF-TC
18	F	29.11	MTLE	TPM, VPA, LTG	1	5	1942.65	2465.73	1765.28	90.9%	6	II	None	None
19	M	16.6	FCD	VPA, LEV, PB	1	5	3049.28	3755.74	2795.58	91.7%	6	III	Temporary decrease in muscle strength	RF-TC

*HH* Hypothalamic hamartoma, *MTLE* Mesial temporal lobe epilepsy, *FCD* Focal cortical dysplasia, *TSC* Tuberous sclerosis complex, *CM* Cavernous malformation, *LGS* Lennox-Gastaut syndrome, *AED* Antiepileptic drugs, *LEV* Levetiracetam, *OXC* Oxcarbazepine, *LTG* Lamotrigine, *LCM* Lacosamide, *VPA* Valproic acid, *TPM* Topiramate, *CBZ* Carbamazepine, *PB* Phenobarbital, *RF-TC* Radiofrequency thermocoagulation

### Patient selection

All patients underwent a thorough preoperative evaluation, which included scalp video electrography (VEEG) monitoring, three-dimensional (3D) 3.0-T magnetic resonance imaging (MRI), functional MRI (fMRI), and diffusion tensor imaging (DTI). In addition, positron emission tomography (PET) and single-photon emission computed tomography (SPECT) were used as auxiliary tests. Some patients also underwent stereotactic electroencephalography (SEEG) and electrode placement for invasive EEG monitoring to help locate the epileptic focus. Imaging data postprocessing was completed once all data acquisition was complete.

Following this evaluation, neurologists and epilepsy surgeons at the epilepsy center discussed all the findings to determine the most effective seizure management method.

The inclusion criteria were as follows: age 6 months to 70 years old, diagnosis of drug-resistant epilepsy, and, because of the nature of the study as a clinical trial involving medical devices, assurance that the patients and their families were fully informed and signed informed consent forms.

The exclusion criteria were as follows: contraindications for the MRI scan, severe coagulation disorders, and pregnancy or lactation.

We usually select patients with a maximum lesion diameter of 2–3 cm for MRgLITT. Although there is no clear upper limit for the lesion size, generally speaking, lesions suitable for complete ablation near the laser probe, i.e., lesions less than 2.5–3 cm, are chosen. For patients with hypothalamic hamartoma (HH), tuberous sclerosis, and cavernous hemangioma, we only performed laser ablation for the lesion itself. For patients with medial temporal lobe epilepsy (MTLE) and FCD, we used SEEG to determine and ablate the susceptible epileptogenic zone. Multiple laser probes can be used for larger lesions, but the possibility of complete ablation is dependent on the combination of probes.

MRgLITT is also applicable for deep focal lesions, including those in HH and MTLE, as opposed to open resection, to reduce the perioperative risk.

The contraindications for MRgLITT include metal implants in the body, severe coagulation dysfunction, and scalp infection.

There are also some patients who have poor epilepsy control as a result of overheated coagulation damage or craniotomy or who require minimally invasive treatment.

### Surgical technique

The target position, size, and probe path trajectory were planned on thin-slice MRI (thickness 1.0 mm, no interval) prior to the operation to avoid important vessels and generate temporary target coordinates. Contrast-enhanced MRI (gadopentetic acid meglumine injection, 0.4 ml/kg once according to body weight) with fiducials determined prior to trajectory planning can allow avoidance of the cortical vasculature when planning the trajectory. Using Huake LITT2.0 software, the target location, size, and probe trajectory were planned out preoperatively on frameless 3D volumetric images, generating temporary target coordinates.

All procedures were carried out with the patient under general anesthesia and the patient’s head fixed with a Mayfield clamp. LITT trajectories were preoperatively planned using the Huake software system. Planning can be performed on the Huakejingzhun mobile workstation and imported into the robotic device. The robotic system is designed to optimize the placement of multiple trajectories efficiently. Both the “probe’s eye view” and “view along the trajectory” were reviewed to confirm that there was no intersection with the vascular structures along the trajectory. A sharp knife was used to cut the skin at the entry point, followed by drilling with a 3.2-mm electric drill. The drilling depth was limited by the skull thickness as measured on preoperative CT. An anchor bolt was screwed into the visible bone, and then, the 1.8-mm laser cooling tube with a metal tube core was carefully inserted to the measured distance. After the laser probe was inserted into the cooling tube, the patient underwent an initial intraoperative MRI scan to confirm the precise placement of the laser probe. Then, laser ablation was performed, and intraoperative MR using near real-time thermal imaging was applied to visualize the system function, the thermal map, and the ablation estimation area. There are two temperature limits on the thermal map: a low-temperature point and a high-temperature point. When the point temperature is exceeded, automatic deactivation of the laser will occur. The low-temperature limit is set to protect functional brain tissue while minimizing damage to surrounding normal tissue and can be set to 45 ~ 50 °C. The high-temperature limit is used to limit the temperature near the applicator to below 90℃ to avoid brain tissue carbonization or evaporation.

**Fig. 1:**
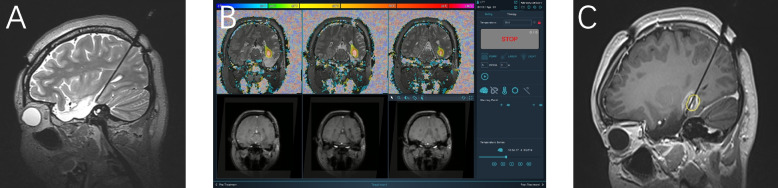
Patient 7: A 24-year-old female still has epileptic seizures after left temporal lobectomy, with approximately 15 seizures per month. It is considered that there is a residual epileptogenic lesion in the medial posterior temporal lobe. **A** Before ablation, T2-weighted interoperative MRI showed a trajectory implanted in the medial posterior aspect of the left temporal lobe. **B** Monitoring planes (coronal position) are determined for the gradient echo scan (GRE) needed for thermal imaging. The high-quality T1-weighted image is overlaid onto the near-real-time thermal and tissue damage calculations. The colored areas in the image represent different temperatures and can be used for real-time temperature measurement. **C** After ablation, T1-enhanced-weighted MRI showed circular enhancement in the posterior temporal lobe, indicating the ablation range

Based on clinical experience, the manufacturer’s recommended settings for laser power were used. Monitoring planes were then determined for the gradient-echo (GRE) scan needed for thermal imaging. High-quality T1-weighted images were overlaid onto the near real-time thermal and tissue damage calculations. The duration of treatment was determined by an MRI damage assessment map, which provided an updated image every 4 s in real-time during ablation. The laser probe was extracted along the puncture needle path within the ablation range as needed to cover a larger area and achieve a wider range of ablation. An example of the entire sequence for a typical patient is shown in Fig. 1.

Following the ablation procedure, an MRI scan was performed to confirm the ablation range. Then, the probe and bone anchor were removed, and the incision was sutured. The patient was extubated and transferred to the ward for overnight monitoring, during which we routinely performed a postoperative CT scan to determine the presence of bleeding.

### Statistical analysis

Demographic, perioperative, and outcome data were collected and recorded. The results of postoperative seizures were evaluated using the Engel epilepsy surgical results scale at the postoperative follow-up (Engel). SPSS 20.0 was used for statistical analysis, which included the *χ*^2^ and Fisher’s exact tests.

## Results

From June 2021 to November 2021, 19 patients in our hospital underwent MRgLITT surgery. Table 1 displays the demographic information of these patients. The average operative age was 18.1 years (range, 3–61.4 years); thirteen patients (68.4%) were male, while six were female (31.6%). These patients took an average of 2.1 antiepileptic drugs. Among the 19 patients, 8 had HH, 5 had MTLE, 3 had focal cortical dysplasia (FCD), 1 had tuberous sclerosis complex (TSC), 1 had cavernous malformation (CM), and 1 had Lennox-Gastaut syndrome (LGS).

Eight patients (8/19; 42%) received treatment with more than one laser fiber, with an average of 1.5 laser fibers per patient (range 1–2). The average postoperative length of stay in the hospital was 4.95 days (range 4–7 days).

Three patients (3/19; 16%) experienced surgery-related complications. One patient experienced a visual field defect, the second patient experienced a minor intracranial hemorrhage, and the third patient experienced a temporary decrease in muscle strength in the left limbs.

At a mean follow-up of 1 year, six patients (6/19; 31.6%) reached Engel class I, 1 patient reached Engel II (1/19; 5.3%), 7 patients reached Engel III (7/19; 36.8%), and 5 patients (5/19; 26.3%) reached Engel class IV. Table 2 describes the clinical characteristics of all patients, including individual Engel classification results.

**Table 2:** Summary of demographic and outcome data

Patient	
Patients, *n*	19
Mean age (range), years	18.1 (3–61.4)
Male, *n* (%)	13 (68.4%)
Female, *n* (%)	6 (31.6%)
Mean AEDs, *n*	2.15
Mean ablation energy, W	8
Mean ablation time, s	2950.5
Mean hospital stay, days	4.95
Complication rate, %	16
Outcome (Engel class), *n* (%)	
Engel I	6 (31.6%)
Engel II + III	8 (42.1%)
Engel IV	4 (21.1%)

*AED* Antiepileptic drugs

Since only 19 patients were in this group, and the total sample size was less than 40, we adopted Fisher’s exact test to analyze the correlation between the Engel classification and epileptogenic disease. We found that this correlation was not statistically significant (*p* = 0.512).

As shown in Table 3, the subgroup analysis was performed to compare a single disease with all other diseases with Engel class I, and the results were not statistically significant.

**Table 3:** Analysis of outcome data by surgical substrate

Surgical substrate	Total, *n*	Engel I	Engel II + III + IV	Fisher’s exact test
Hypothalamic hamartoma	8	2	6	0.341
Focal cortical dysplasia	3	1	2	0.483
Tuberous sclerosis	1	1	0	0.316
Mesial temporal sclerosis	5	1	4	0.369
Cavernous malformation	1	1	0	0.316
LGS	1	0	1	0.684

*LGS* Lennox-Gastaut syndrome

## Discussion

### Efficacy analysis

Based on our findings, the rate of postoperative epilepsy control in the 19 patients was high. Following the operation, 6 patients had no seizures during follow-up, and the rate of seizures in the vast majority of patients decreased significantly.

Among these patients, 8 with hypothalamic hamartoma experienced varying degrees of postoperative relief in their epilepsy episodes, with 2 patients achieving seizure-free status. LITT for hypothalamic hamartoma has a better effect on controlling gelastic seizures than open surgery or radiation therapy [3, 4]. One study revealed that adequate epilepsy control can be achieved even without complete ablation of the focus, e.g., with approximately half of the ablation volume. Large hamartomas require multiple laser treatments to achieve safe and phased disconnection of the lesion without causing damage to the normal brain. Functional disconnection through a bilateral path is usually not required for HHs with bilateral connections [5].

In the treatment of MTLE, MR-guided LITT (MRgLITT) is becoming a viable alternative to open temporal lobe surgery, particularly in patients with hippocampal sclerosis. The postoperative seizure-free rate of craniotomy for anterior temporal lobe and medial structure resection is approximately 58% [6]. After open surgery, the seizure-free rate can reach 60–90% [7], while LITT can achieve a seizure-free rate of 55% [8]. LITT has also demonstrated a higher percentage of Engel class I in patients with medial temporal lobe atrophy [9].

Corpus callosotomy can be used to treat generalized epilepsy in children and adults, particularly those with tonic and atonic seizures [10]. Full-length incisions are preferred in children [11], whereas in adults, a 2/3 incision can be considered first. For the lone adult patient in our group with LGS, we performed anterior corpus callosotomy with LITT. DTI can be used to confirm that the fractional anisotropy (FA) and average diffusion coefficient (ADC) of the corpus callosum fiber bundle projection have decreased significantly after the operation [12]. Double-fiber implantation in the frontal and parietal lobes allows for a 2/3 anterior-segment incision in the corpus callosum as well as an increase in the ablation range of the incision [13]. The effect in terms of epilepsy control was poor at the time of reexamination 3 months after the operation, and many seizures were still occurring. Huang and colleagues reported the treatment of six patients by LITT for complete corpus callosum incision in 2019. During the follow-up period, three adults reached Engel classes I–II, and three children reached classes III–IV [14].

CM resection guided by cortical EEG can achieve an epilepsy control rate of 70–80%. Of course, craniotomy carries some additional risks, particularly in the treatment of deep lesions or those located in functional areas. LITT, on the other hand, can achieve an Engel class I percentage of 82% [15]. Our CM patient had no seizures after the operation.

After craniotomy for frontal lobe epilepsy, the overall seizure-free rate is approximately 57.1%. From 6 months to 5 years, the seizure-free rate decreases from 67 to 46%. Within the first year after surgery, 83% of patients experience epilepsy recurrence [16]. In a 2015 study, 17 children with intractable epilepsy, including 11 with FCD, underwent LITT. Within an average follow-up of 16.1 months, 7 patients achieved Engel class I, accounting for 63.6% of the patients [17]. In our study, there were 3 patients with FCD, of whom 1 had no postoperative episodes, while the other 2 had significantly reduced postoperative episodes. LITT is regarded as a safe and effective option for treating seizures without requiring a craniotomy [18, 19].

### Safety analysis and cautions on the operation

The main advantage of this operation is its safety. According to the statistical analysis, the average amount of bleeding during the procedure was approximately 7.26 ml, and the average ablation time was approximately 49 min. The correlation between the ablation range determined by comparing the postoperative MRI findings and the preoperative plan was approximately 92.0%, indicating high accuracy and ensuring a postoperative effect. Furthermore, the average length of stay in the hospital for the patients was 4.94 days, including a patient with minor bleeding after the operation. The condition of patients with bleeding should be monitored until the bleeding is confirmed to be stable 7 days after the operation. This approach significantly reduces the duration and costs of hospitalization. According to one study, 89% of patients with HH treated with LITT were hospitalized for only one night [3].

In our study, 5 cases of MTLE were treated with double-fiber implantation, the first of which was inserted into the amygdala from the hippocampal head and the second of which was used to ablate the body and tail of the hippocampus along its long axis. We believe that this double-fiber configuration can cover the curved anatomical structure of the medial temporal lobe, allowing for a wider ablation range and a reduction in residual epileptogenic lesions, resulting in a better effect. Residual medial hippocampal head tissue after LITT ablation has been found to be significantly related to persistent postoperative seizures [20], and ablation through the puncture channel on the side of the hippocampus is also associated with a poor prognosis [20].

One patient diagnosed with TSC in our group underwent ablation after preoperative multimodal imaging and electrophysiological evaluation, with no seizures after the procedure. The age of onset for patients with tuberous sclerosis is frequently very low; the skulls of children are thin, and thus, there is a high risk of penetrating the skull during head fixation. LITT has stringent navigation accuracy requirements, which complicate navigation registration. One study reported the use of frameless navigation technology, with a minimum age for LITT reaching down to 6 months with the help of a microtripod system and an intraoperative reference point [21]; however, the number of reported cases is very low [22].

During corpus callosotomy with LITT, we found that when the optical fiber was inserted from the parietal lobe, because the target point is almost horizontal and the drill bit cannot be applied vertically with the bone plate, it enters at an oblique angle, causing deflection during implantation, especially when the implantation path is long, as a slight deflection of the entry point causes greater deviation from the target point. In our experience, the bit holder should be as close to the bone plate as possible when drilling to minimize deviations during drilling. Double-fiber implantation at an angle can be used to ablate additional tissue ranging from the knee to the beak of the corpus callosum. Of course, the longer the optical fiber implantation path, the more likely complications become. During implantation, intraventricular choroids and blood vessels should be considered. More advanced computer-aided planning systems can receive imaging data into an automatic matching algorithm and provide doctors with multiple paths from which to choose; this approach allows complete ablation to be achieved more safely and efficiently, avoiding residual lesions and reducing postoperative seizures [23].

The difficulty with a cavernous hemangioma is that it is difficult to implant an optical fiber into the lesion due to its hard and tough texture, resulting in target deviation. Willie et al. investigated three patients with cavernous hemangioma following LITT for craniotomy and epileptogenic area resection. The pathological findings revealed reactive astrocyte proliferation and hemosiderin-containing macrophages, indicating a history of hemorrhagic disease and inflammatory cell infiltration rather than the typical manifestations of cavernous vascular malformation. LITT is thought to be capable of fully ablating the cavernous hemangioma tissue structure due to scattered calcification and obvious calcified fibrous/sclerotic arachnoid tissue [15]. From the standpoint of safety, the risk of bleeding caused by optical fiber puncture is low. First, the internal blood vessels in cavernous hemangioma have thin walls and low pressure, making bleeding difficult. Second, experiences with craniotomy have shown that if the relevant blood vessels are fully protected, bleeding is unlikely [24].

One patient with HH in this group had undergone SEEG-guided radiofrequency TC (RF-TC). Following the ablation, severe diabetes insipidus, electrolyte disorder, high fever, excessive appetite, and generalized tonic–clonic seizures occurred, gradually stabilizing after nearly 2 months of comprehensive treatment. During the operation, we referred to the stereotactic SEEG RF-TC and used Sinovation v2.0 software developed by Huake for the SEEG/LITT program, which can simulate the volume of the probe after ablation and reconstruct the volume through multimodal 3D imaging to help visualize the structures and lesions to be treated to make multiple optical fibers reach the target from different directions, forming a spatial combination and achieving maximum volume ablation. Due to the real-time detection and power time controllability of LITT, it is more flexible than RF-TC. Even in minimally invasive treatment, RF-TC carries a risk of serious complications due to the lack of intraoperative MR monitoring. Although our data are insufficient to explain the significant difference, LITT has unrivaled advantages in the management of complications.

### Complication analysis

Three patients had complications as a result of the operation. One of these patients had temporal lobe epilepsy. Prior to the operation, the patient underwent a craniotomy to remove a portion of the medial temporal lobe and implant an optical fiber. After the operation, it was discovered that the position was slightly lateral to the target, and there was isotropic hemianopia of the binocular visual field. This symptom had resolved on reexamination 3 months after the operation. It is thought that injury to Meyer’s loop posterolateral to the ablation range resulted in optic radiation injury. Visual impairment, including test–retest variability and visual field deficit, is the most common postoperative complication of LITT in the treatment of MTLE, with an overall incidence of 5.1% [25]. One of our enrolled patients experienced a visual field defect, i.e., bilateral isotropic hemianopia, after surgery. This case may have been related to the external position of optical fiber implantation and damage to the optic bundle or Meyer loop structure during ablation. Of course, this is a common complication in open surgery, occurring in 6% of patients [26]. There is also the possibility of neurocognitive impairment [27]. Postoperative naming disorder on the dominant side and facial recognition disorder on the nondominant side are significantly reduced in LITT. However, there is no difference in postoperative memory decline compared to traditional open surgery or in surgical complications such as intracerebral hemorrhage, subarachnoid hemorrhage, subdural hemorrhage, visual field deficit, and cranial neuropathy [9].

Another patient had right temporal occipital lobe FCD, which had previously been resected. During CT reexamination after ablation, it was discovered that there was a small amount of bleeding in the brain parenchyma of the operative area, which did not cause symptoms. Because axial thermocoagulation was performed along the puncture tract during the procedure, it was impossible to determine whether the bleeding was due to the puncture or ablation. The blood had been absorbed by CT reexamination of the head on the sixth day after surgery. These first two cases may have been related to the longer puncture path for optic fiber implantation and the involvement of more important pathway structures.

The third case involved a right frontal lobe FCD lesion, and because the lesion was close to the primary motor cortex, there was a temporary decrease in muscle strength in the left limb following the operation. This complication was anticipated prior to the operation, and the procedure was carried out after extensive consultation with the patient and his family members. After understanding the risk, the patient agreed to the operation, and the loss of muscle strength gradually improved postoperatively. Before discharge, the muscle strength had returned to the preoperative level. This complication was related to the surgical site.

This study represents the first batch of case data to be included in the evaluation of the preliminary treatment characteristics of LITT, but our study is also limited by the sample size being too small to allow statistically significant conclusions to be drawn. Furthermore, our results were obtained over a short follow-up period, and the evaluation of the results could also have been influenced by the imaging method. Thus, we need to continue to follow up with this group of patients for a longer period of time to be able to draw more accurate conclusions.

## Conclusion

As a minimally invasive method for the ablation of drug-resistant epileptic lesions, MRgLITT has high safety, a low complication rate, and a good surgical effect. It may even be used as a partial replacement for craniotomy in the future. However, due to the limitations of this study, a larger, multicenter, prospective MRgLITT study with a longer follow-up period is still required to improve the findings.

## Data Availability

All data generated or analyzed during this study are included in this published article.

## Abbreviations

AED: Antiepileptic drugs
CBZ: Carbamazepine
CM: Cavernous malformation
CT: Computed tomography
DTI: Diffusion tensor imaging
FCD: Focal cortical dysplasia
HH: Hypothalamic hamartoma
LCM: Lacosamide
LEV: Levetiracetam
LGS: Lennox–Gastaut syndrome
LITT: Laser interstitial thermal therapy
LTG: Lamotrigine
MRgLITT: Magnetic resonance (MR)-guided LITT
MRI: Magnetic resonance imaging
MTLE: Mesial temporal lobe epilepsy
OXC: Oxcarbazepine
PB: Phenobarbital
PET: Positron emission tomography
RF-TC: Radiofrequency thermocoagulation
SPECT: Single-photon emission computed tomography
SEEG: Stereotactic electroencephalography
TPM: Topiramate
TSC: Tuberous sclerosis complex
VEEG: Video electrography
VPA: Valproic acid
